# The reactivity of acyl chlorides towards sodium phosphaethynolate, Na(OCP): a mechanistic case study†
†Electronic supplementary information (ESI) available. CCDC 986673, 1454789 and 1454790. For ESI and crystallographic data in CIF or other electronic format see DOI: 10.1039/c6sc01269h


**DOI:** 10.1039/c6sc01269h

**Published:** 2016-06-17

**Authors:** Dominikus Heift, Zoltán Benkő, Riccardo Suter, René Verel, Hansjörg Grützmacher

**Affiliations:** a Department of Chemistry and Applied Biosciences , ETH Zurich , CH-8093 Zurich , Switzerland . Email: hgruetzmacher@ethz.ch ; Email: zbenko@mail.bme.hu; b Lehn Institute of Functional Materials (LIFM) , Sun Yat-Sen University , 510275 Guangzhou , China; c Budapest University of Technology and Economics , H-1111 Budapest , Hungary

## Abstract

The reaction of Na(OCP) with mesitoyl chloride delivers an ester functionalized 1,2,4-oxadiphosphole in a clean and P-atom economic way. The reaction mechanism has been elucidated by means of detailed NMR-spectroscopic, kinetic and computational studies. The initially formed acyl phosphaketene undergoes a pseudo-coarctate cyclization with an (OCP)^–^ anion under the loss of carbon monoxide to yield a five-membered ring anion. Subsequently, the nucleophilic attack of the formed heterocyclic anion on a second acyl chloride molecule results in the 1,2,4-oxadiphosphole. The transient acyl phosphaketene is conserved during the reaction in the form of four-membered ring adducts, which act as a reservoir. Consequently, the phosphaethynolate anion has three different functions in these reactions: it acts as a nucleophile, as an en-component in [2 + 2] cycloadditions and as a formal P^–^ transfer reagent.

## Abstract 2

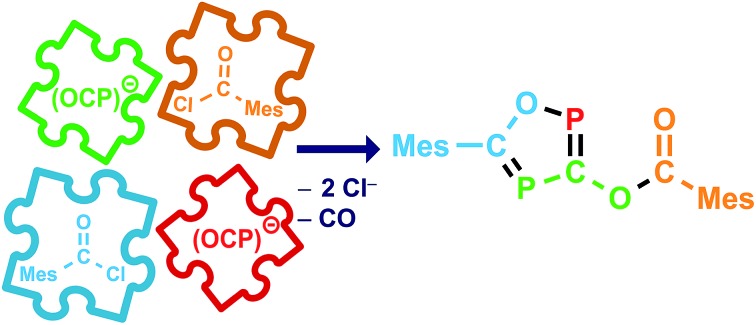
The phosphaethynolate anion (OCP)^–^ has three different functions in the reaction with 2,4,6-trimethylbenzoyl chloride (MesCOCl): it acts as a nucleophile, as an en-component in [2 + 2] cycloadditions and as a formal P^–^ transfer reagent.

## Introduction

**Fig. 1:**
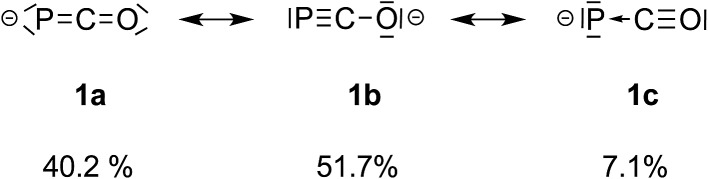
Resonance structures of the phosphaethynolate anion.

Decarbonylation reactions, such as the transformation of aldehydes^1^ into hydrocarbons, are widely utilized for the removal of carbonyl groups in organic syntheses.^2^ As special congeners of carbonyl compounds, ketenes can undergo photochemical decarbonylation, yielding carbenes as reactive species.^3^ Analogously, decarbonylation reactions of phosphaketenes R–P=C=O have been described.^4^ These photochemical^5^ or transition metal assisted^6^ reactions deliver – *via* the cleavage of the PC double bond – transient phosphinidenes or phosphinidene complexes, respectively. Recently, we reported the synthesis of several hetero-phosphaketenes employing the phosphaethynolate (OCP)^–^ anion as a phosphorous nucleophile. Furthermore, the ambident character of this anion has been demonstrated.^7^ According to the analysis of the natural resonance structures, the weighting of the phosphaethynolate mesomeric structure **1b** is slightly larger than that of **1a** (Fig. 1).7*b* Besides these two predominant structures, though with a much smaller weighting, structure **1c** contributes to the electronic ground state. It describes the (OCP)^–^ anion as a donor–acceptor complex of a P^–^ ion and carbon monoxide. Similar to the description of a transition metal carbonyl complex, the CO unit acts as a σ-donor and a π-acceptor.

The delocalization of the negative charge hampers the spontaneous decarbonylation of the (OCP)^–^ ion, however, the contribution of the mesomeric structure **1c** suggests that this anion may act as a (formal) P^–^ transfer reagent. Indeed, we could demonstrate that the reaction of an imidazolium salt with Na(OCP) forms the adduct of the parent phosphinidene (P–H) with the corresponding N-heterocyclic carbene.^8^ According to the proposed mechanism, the parent phosphaketene (H–P=C=O) is formed as an intermediate, which delivers the PH fragment in a concerted reaction step under the extrusion of CO.^9^ The (OCP)^–^ anion was found to be a useful synthon to obtain heterocycles and cages,^10^ especially when accompanied by the cleavage of the C–P bond.^11^ In general, in this way, anionic phosphorous heterocycles are accessible and there are two possibilities: (i) the CO moiety can still be incorporated in the final product as a carbonyl group or (ii) carbon monoxide may be released in the reaction as a gaseous by-product. When Na(OCP) is reacted with two equivalents of a small carbodiimide^12^ or an activated alkyne (PhC≡C–CO_2_Et),11*a* substituted six-membered phosphorous heterocyclic anions bearing the P atom and the carbonyl functionality in the 1,4-position are formed. However, with the more reactive diethyl acetylenedicarboxylate, besides the analogous six-membered ring, a substituted phospholide [PC_4_(COOEt)_4_]^–^ was also obtained, accompanied by the loss of carbon monoxide.11*a* In a similar reaction, (OCP)^–^ and two equivalents of isocyanate formed five-membered azadiphospholides, which were found to be active catalysts for isocyanate trimerization *via* spiro phosphoranides.^13^ Furthermore, the phosphorous analogues of isocyanates, phosphaketenes, were also reacted with Na(OCP), resulting in triphospholides in a cycloaddition–decarbonylation process in which short-lived intermediates were identified using low-temperature NMR spectroscopy for the first time.^14^ In order to understand the reaction pathways leading to these heterocycles, significant computational efforts have been made,11c,^12^–^15^ but the decarbonylation process still lacks a fundamental understanding.

Here, we report the results of our investigations on the reactivity of Na(OCP)11c,^16^ towards acyl chlorides. We provide the mechanistic understanding of a rather complex but selective reaction, which employs the (OCP)^–^ anion as both P nucleophile and P^–^ transfer reagent.

## Results and discussion

**Scheme 1:**
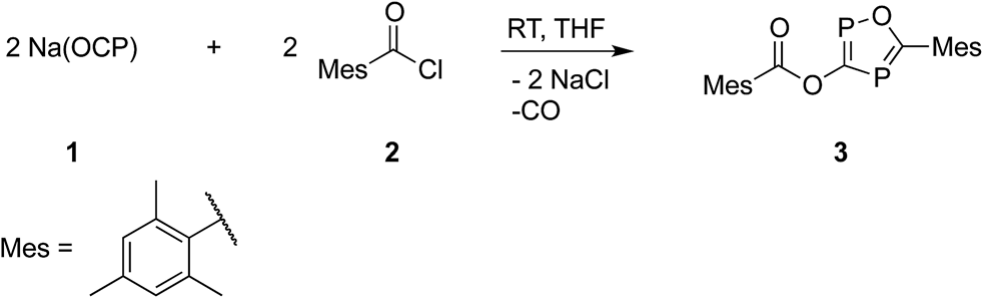
Formation of the acyl substituted 1,2,4-oxadiphosphole **3**.

Initially, we attempted to synthesize acyl phosphaketenes RC(O)(P=C=O), which are the heavier analogues of acyl isocyanates. Knowing that sterically demanding groups are needed to stabilize a phosphaketene in a monomeric form,^17^ in the first experiment Na(OCP)(dioxane)_2.5_ was reacted with the rather bulky 2,4,6-trimethylbenzoyl chloride, MesCOCl, in a 1 : 1 ratio (Scheme 1). The precipitation of sodium chloride and a little gas evolution was observed during the reaction. Subsequent investigation of the reaction solution using ^31^P-NMR spectroscopy showed no resonance in the expected chemical shift range for a mesitoyl phosphaketene (note that the calculated chemical shift of MesCO(PCO) is –110 ppm at the B3LYP/aug-cc-pVDZ level of theory). Instead, two doublet resonances at *δ* = 112 and 253 ppm with a coupling constant of *J*_PP_ = 46 Hz were observed. The ^13^C-NMR spectrum and in addition X-ray diffraction analysis revealed the formation of the acyloxy substituted 1,2,4-oxadiphosphole **3** (Scheme 1), which was isolated as a light sensitive, yellow solid in good yield (70%).

There are only a handful of synthetic procedures for 1,2,4-oxadiphospholes, which afford only poor to moderate yields.^18^ These methods usually involve several reaction steps and complicated techniques are needed to separate the desired product from large amounts of by-product. The reaction of the easily accessible Na(OCP) with MesCOCl offers a straightforward synthesis with a good atom economy with respect to phosphorous.^19^

**Fig. 2:**
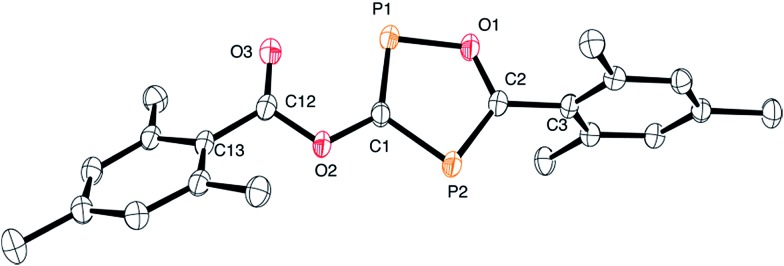
ORTEP plot of **3** (thermal ellipsoids are drawn at 50% probability). Hydrogen atoms have been omitted for clarity. Selected bond lengths (Å) and angles (°): P1–C1 1.716(3), C1–P2 1.780(2), C2–P2 1.715(2), C2–O1 1.346(3), O1–P1 1.696(2), C2–C3 1.497(3), C1–O2 1.395(3), C12–O3 1.219(3), C12–C13 1.489(3); C1–P1–O1 93.0(1), P1–O1–C2 118.7(2), O1–C2–P2 119.2(2), C2–P2–C1 90.9(1), C1–O2–C12 116.0(2), O2–C12–C13 112.7(2), O3–C12–C13 127.0(2).

The structure of **3** is shown in Fig. 2. The oxadiphosphole ring and the attached ester moiety are coplanar, while the mesityl rings are rotated out of this plane by 44.2 and 84.5°. The PC and CO bond lengths of the P_2_C_2_O ring are comparable to those in other 1,3-oxaphospholes.^20^

**Fig. 3:**
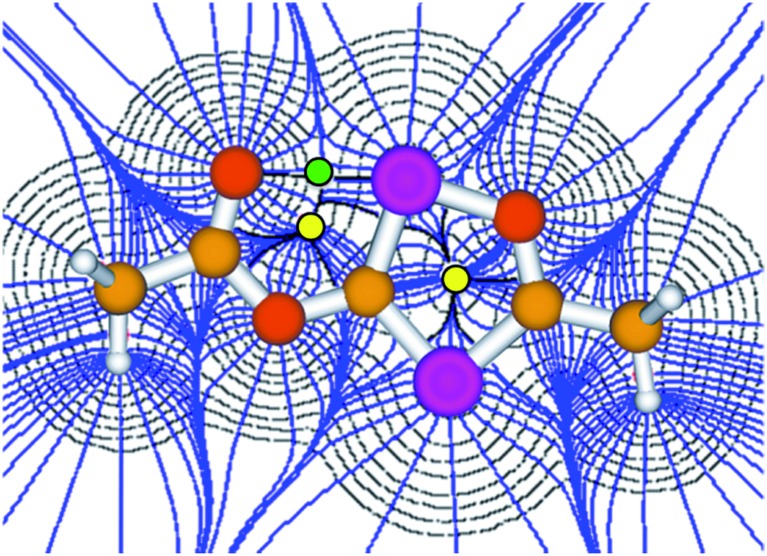
Atoms in molecules (AIM) analysis of **3′** at the B3LYP/6-311+G** level of theory. The green and yellow dots represent the bond and ring critical points, respectively.

Although the P–C bond lengths indicate a Lewis valence electron distribution with alternating single (C1–P2) and double bonds (C1–P1 and C2–P2), some extent of bond length equalization can be observed, which indicates a moderate aromatic delocalization. This is in agreement with previous theoretical studies, which stated that the aromaticity of the parent 1,2,4-oxadiphosphole is just slightly smaller than that of furan and other phosphasubstituted furans.^21^ Nuclear independent chemical shift^22^ calculations revealed that the ester substituent has only a very small influence on the aromaticity of the five-membered ring.^23^ A striking feature of the structure shown in Fig. 2 is the rather short P1–O3 distance (2.5 Å), which is significantly smaller than the sum of the van der Waals radii of phosphorous and oxygen (3.32 Å),^24^ indicating a secondary interaction. Note that the oxygen atom O3 of the carbonyl group faces the phosphorous atom P1, which is positively polarized by the neighboring O1 atom. A topological analysis^25^ of the electron density on a model system **3′** (methyl instead of mesityl groups) shows a bond critical point between P1 and O3 (see Fig. 3). The corresponding electron density of *ρ* = 0.029 a.u. indicates a relatively strong interaction in the range of hydrogen bonds.

Since *four* equivalents of reactants are involved [2 MesCOCl + 2 Na(OCP)], a rather complex reaction mechanism with a number of steps must be involved in the formation of **3**. To explore the reaction sequence, low temperature NMR measurements were performed. At room temperature the reaction is complete within mixing time of the reagents. A THF solution containing stoichiometric amounts of the starting materials was gradually warmed from –50 °C to room temperature and the progress of the reaction monitored using ^31^P-NMR spectroscopy. At –35 °C the parallel formation of two intermediates **A** and **B** was observed. NMR investigations at different temperatures indicated that **B** is in equilibrium with **A** and an (OCP)^–^ anion. Intermediates **A** and **B**, the final product **3** and Na(OCP) are the only species which can be detected using ^31^P-NMR spectroscopy. NMR experiments using different stoichiometric ratios of the starting materials did give the same results.

**Fig. 4:**
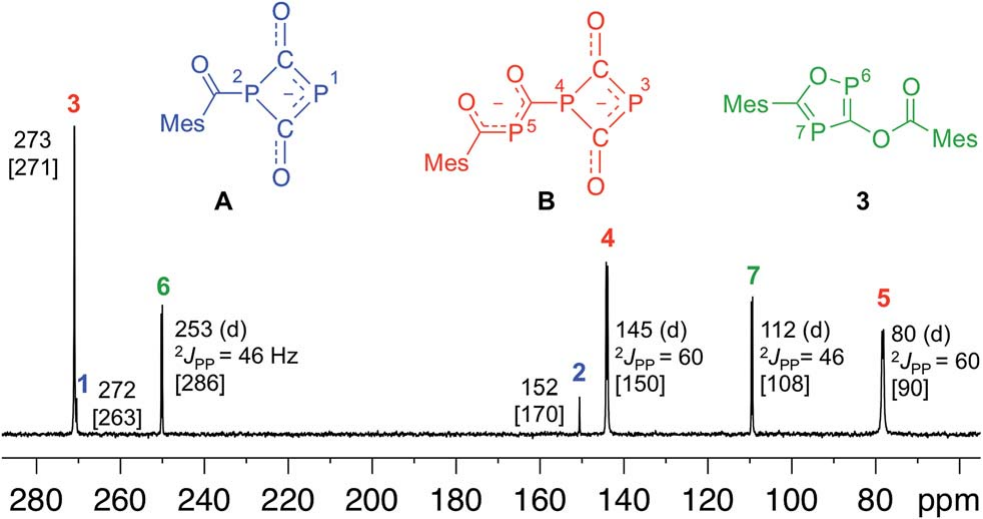
^31^P-NMR spectrum at –35 °C after 17 h reaction time showing intermediates **A**, **B** and the final product **3**. Chemical shifts are given in ppm, coupling constants (*J*) in Hz. Calculated chemical shifts at the B3LYP/aug-cc-pVDZ level are given in brackets.

Anion **A** and dianion **B** were identified using GIAO chemical shift calculations, which are shown in Fig. 4 together with the experimental data. **A** and **B** are adducts of a mesitoyl phosphaketene molecule with one and two (OCP)^–^ ions, respectively. This indirectly indicates the initial formation of mesitoyl phosphaketene, MesCO-P=C=O. The common structural motif of **A** and **B** is the four-membered P(CO)_2_P ring, which was first described for [O_2_C–P(CO)_2_P]^2–^, the adduct of a CO_2_ molecule and two (OCP)^–^ ions.11*c* The ^31^P-NMR chemical shifts of this adduct (*δ* = 279 and 102 ppm) are indeed very similar to those of the rings in **A** and **B**. The ^31^P-NMR chemical shift of the P5 (80 ppm) atom in **B** matches well with that of bismesitoyl phosphide [P(COMes)_2_]^–^, (*δ* = 86 ppm).^26^

**Scheme 2:**
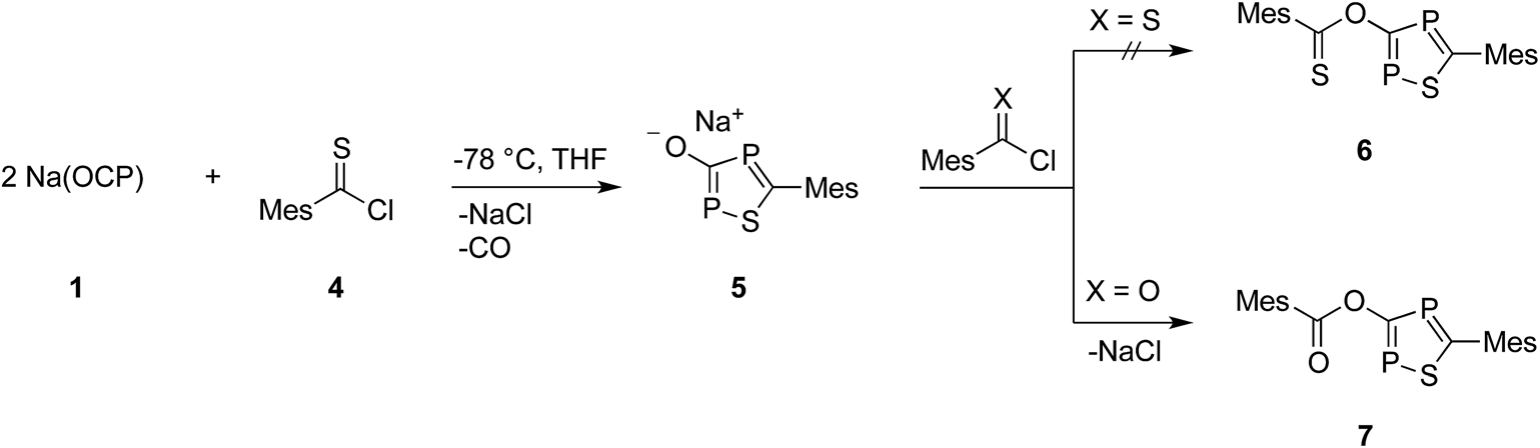
Formation of sodium 1,2,4-thiadiphosphol-3-olate (**5**) and its reaction with (thio)acyl chloride.

**Fig. 5:**
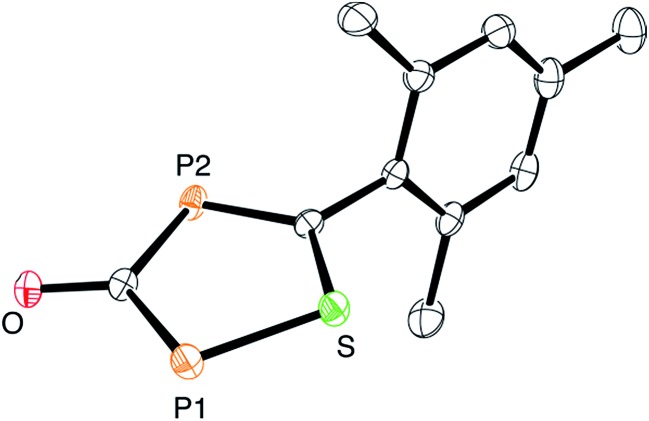
ORTEP plot of **5** (thermal ellipsoids are drawn at 50% probability). The sodium counter ion with two coordinated DME molecules and hydrogen atoms have been omitted for clarity.

At this point, it is not obvious how the intermediates **A** and **B** are involved in the formation of **3**. We therefore reacted 2,4,6-trimethylbenzothioyl chloride (**4**) with Na(OCP) in the hope of identifying another possible intermediate. When Na(OCP) and **4** were reacted at –78 °C in a ratio of 2 : 1, again gas evolution indicated the formation of carbon monoxide. Using NMR spectroscopy we could observe the formation of the anion **5**, which was isolated in low yield (Scheme 2). The structure of **5** with a sodium counter ion was unambiguously determined using single crystal X-ray diffraction analysis (see Fig. 5).

Compound **6**, which is the disulfur analogue of **3**, was not formed even when thioacyl chloride was used in excess. However, when **5** was reacted with mesitoyl chloride, the 1,2,4-thiadiphosphole **7** was obtained as the monosulfur analogue of **3**.^27^ The outcome of these experiments is in line with the lower electrophilicity of thioacyl chlorides when compared to acyl chlorides. Importantly, these results also give further hints to a possible reaction mechanism for the formation of oxadiphosphole **3**. We assume that in the last step a mesitoyl chloride molecule reacts with a 1,2,4-oxadiphosphol-3-olate anion, which is the oxygen analogue of **5**. However, this anion was not observed in the ^31^P-NMR spectra, which implies that it rapidly reacts further with acyl chloride.

**Scheme 3:**
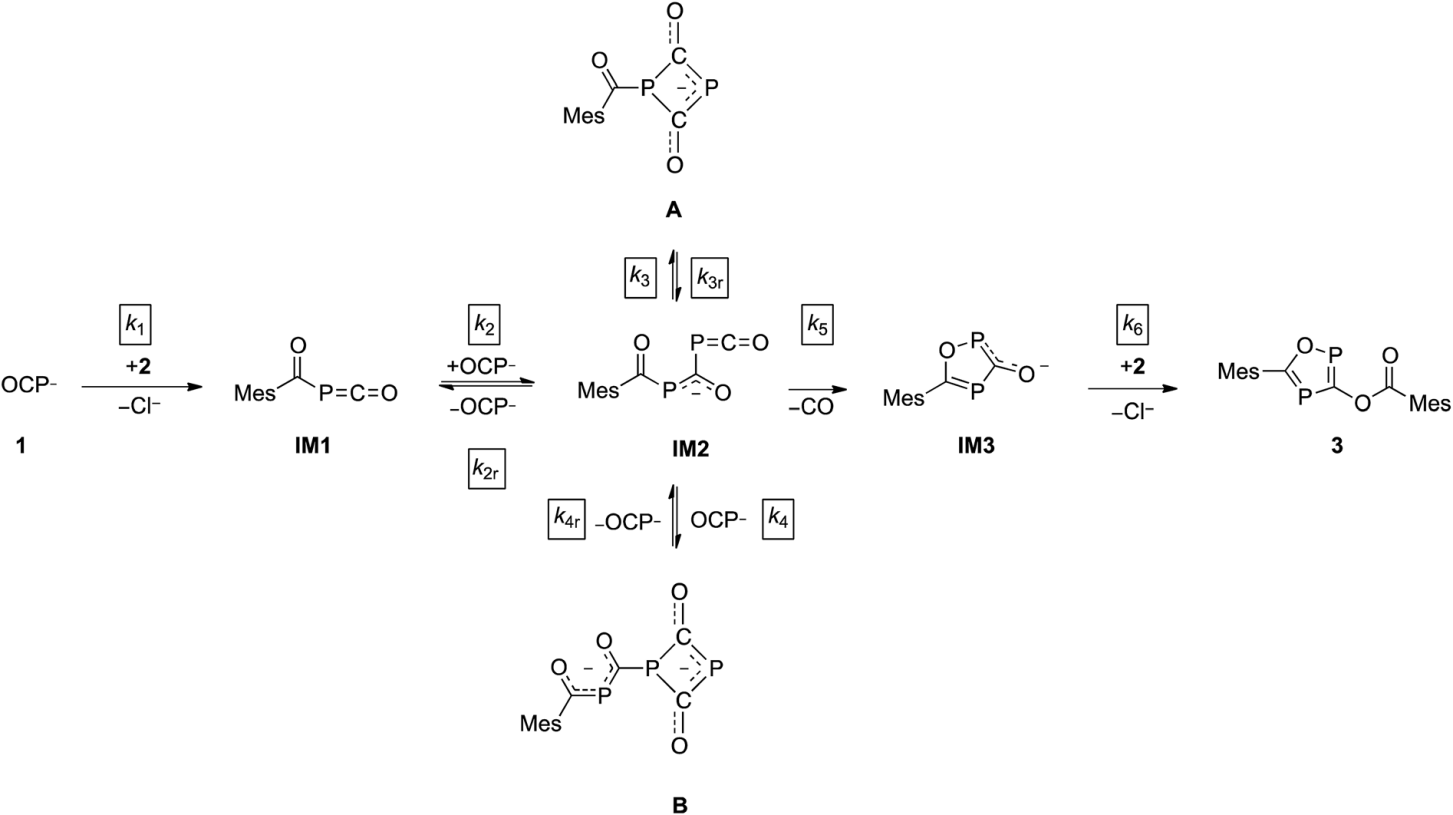
Proposed reaction mechanism leading to **3**.

The direct observation of intermediates **A** and **B**, as well as the indirect evidence for the 1,2,4-oxadiphosphol-3-olate anion, allowed us to propose a reaction sequence for the formation of **3** which is shown in Scheme 3.

In the first step, the acyl phosphaketene forms as intermediate **IM1**. This is subsequently attacked by an (OCP)^–^ ion, leading to **IM2**. From intermediate **IM2**, which was not observed experimentally, the cyclic anions **A**, **B** and **IM3** can be formed. A [2 + 2] ring closure of **IM2** leads to its isomer **A**, while a formal cycloaddition of **IM2** with another equivalent of (OCP)^–^ anion delivers intermediate **B**. Presumably, the reactions leading from **IM1** to **IM2** as well as from **IM2** to the intermediates **A** and **B** are reversible. This is supported by the experimentally observed equilibrium between **A**, an (OCP)^–^ anion and **B** (*vide supra*).^28^ Furthermore, **IM2** can undergo a cyclization under the loss of CO to yield **IM3**. In the final step, the nucleophilic substitution on an acyl chloride with **IM3** gives the final product **3**.

**Fig. 6:**
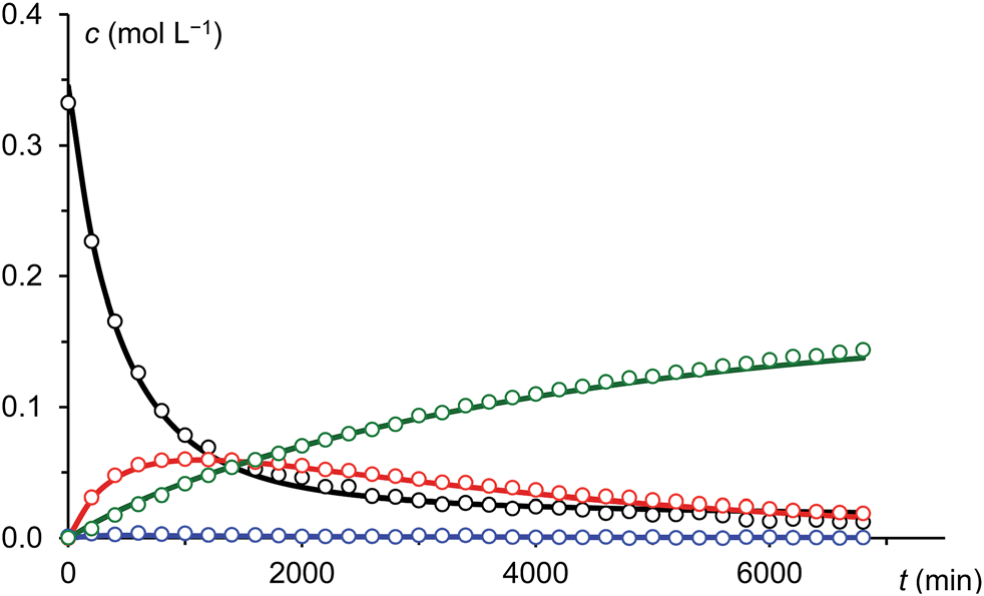
Measured (dots) and fitted (solid lines) concentrations of Na(OCP) (black), **A** (blue), **B** (red) and **3** (green).

Under the assumption that the reaction mechanism shown in Scheme 3 is correct, a kinetic study was carried out in THF as solvent. The reaction progress was followed at –35 °C using ^31^P-NMR spectroscopy and the concentrations of Na(OCP), **A**, **B** and the final product **3** were determined from the relative integrals of the corresponding peak areas (see Fig. 6). Applying non-linear fitting to these data, the rate constants for the reaction steps shown in Scheme 3 were estimated (see Table 1). As certain species could not be included in the fitting (either they are not observable on the NMR time scale or do not contain P atoms), this method cannot exclude the possibility of other (slightly different) reaction pathways. However, the fitted model describes the concentration time dependency of the reaction system reasonably well and gives rate constants within a sensible range.

**Table 1:** Estimated rate constants (for first order reactions in min^–1^, for second order in L × mol^–1^ × min^–1^), see Fig. 6. The last step of the reaction is instantaneous, thus *k*_6_ is assumed to be much faster than the others and is not listed

Forward reaction		Reverse reaction	
*k*_1_	2.36 × 10^–3^		
*k*_2_	4.98	*k*_2r_	1.47
*k*_3_	2.08 × 10^–2^	*k*_3r_	5.02 × 10^–3^
*k*_4_	8.40 × 10^–1^	*k*_4r_	3.64 × 10^–4^
*k*_5_	8.19 × 10^–2^		

In this model, the nucleophilic substitution of Cl^–^ in mesitoyl chloride for (OCP)^–^ is slower than the subsequent reaction of the activated acyl phosphaketene MesCO–P=C=O **IM1** with another equivalent of (OCP)^–^. Indeed, this reaction between **IM1** and (OCP)^–^ is the fastest of all and explains why **IM1** is not observed. Intermediate **IM2** – likewise not observed – is consumed by four competing reactions: the back reaction to **IM1** and (OCP)^–^, the reversible formation of **A**, the reversible formation of **B**, which is faster than that of **A** (*i.e. k*_4_ > *k*_3_), and the irreversible formation of **IM3** which is faster than that of **A** (*i.e. k*_5_ > *k*_3_) but smaller than that of **B** (*i.e. k*_5_ < *k*_4_). That **A** and **B** are transient but observable species is due to the slow back reaction to **IM2**, especially for **B** → **IM2** which has the smallest rate constant, *k*_4r_. Consequently, the concentration of **B** increases steeply at the beginning of the reaction (in the first *ca.* 15 hours) and serves as a reservoir for intermediate **IM2**. Similarly, **A** is a conserved form of **IM2**, however in this case the accumulation effect is much less pronounced (*k*_3_ is similar to *k*_3r_) and the concentration of **A** is low and remains approximately constant throughout the reaction. **IM2** is consumed irreversibly under loss of CO to give **IM3**, which rapidly and irreversibly reacts with acyl chloride to give the final product **3**.

**Fig. 7:**
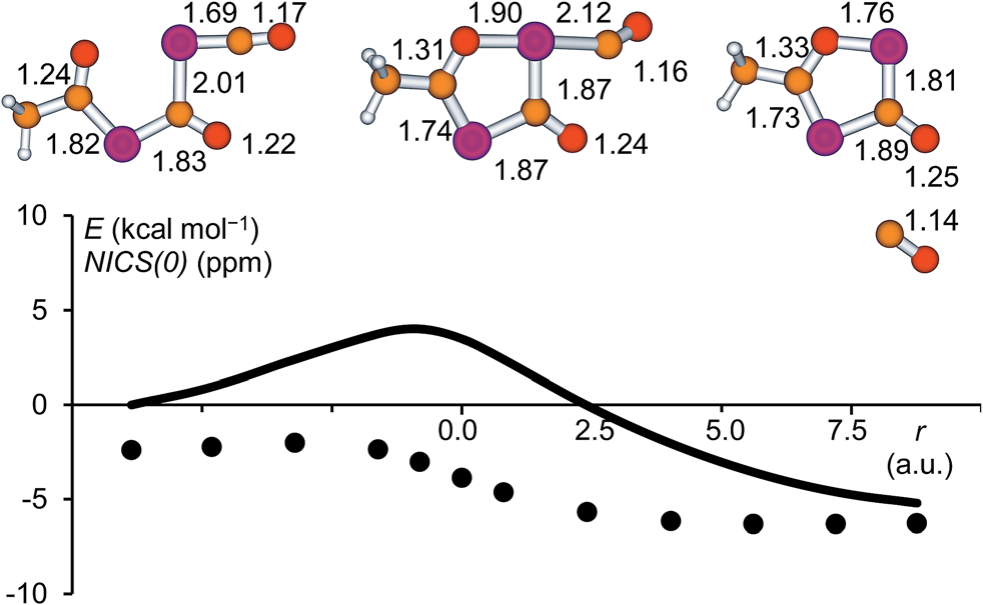
Relative energy (solid line, CCSD(T)/aug-cc-pVDZ//B3LYP/6-31+G*) and NICS(0) values (dots, B3LYP/6-311+G**//B3LYP/6-31+G*) along the reaction coordinate for the ring closure reaction leading from **IM2** to **IM3**. Selected atom distances are given in Å for **IM2**, the transition state and **IM3**.

In the present work, the reactions leading from **IM2** to **A** and **IM2** to **B** were not investigated computationally, since similar transformations were subjects of a previous study.^28^ The formation of **IM3** from **IM2**, however, deserves a closer look. High-level calculations revealed that this reaction follows a concerted mechanism and the activation barrier was found to be low (4.0 kcal mol^–1^ at the CCSD(T)/aug-cc-pVDZ//B3LYP/6-31+G* level of theory). The structure of the corresponding transition state is shown in Fig. 7 and the geometrical parameters indicate a late transition state. The cleavage of the P=C double bond of the phosphaketene moiety occurs simultaneously with the formation of the P–O bond. A continuous change in the atom distances was observed along the reaction coordinate. In this process, the starting material, which consists of a phosphaketene unit and a separate delocalized OCPCO moiety, transforms to a moderately aromatic system. The increase in aromaticity is reflected by the NICS(0) values depicted as a function of the reaction coordinate, which become continuously more negative.

**Fig. 8:**
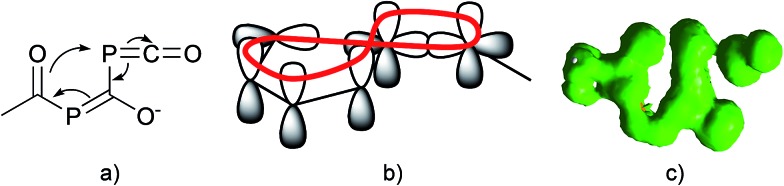
(a) Coarctation at the P atom of the phosphaketene unit; (b) Möbius type eight-electron interaction; (c) ACID plot of the transition state (isosurface value: 0.020 a.u.) at the CGST/B3LYP/6-31+G* level.

Since there is an exocyclic part involved in this bond transformation process, we propose that this reaction is of coarctate type. Besides classical linear and pericyclic reactions, a third group of reactions was classified by Herges as coarctate.^29^ Coarctate (or complex) reactions proceed in a concerted manner by breaking and making two bonds at one (or more) atoms at a time. In a pericyclic reaction the bond making and bond breaking occurs simultaneously in a cyclic pathway. On the contrary, the coarctate transition state exhibits a so-called coarctate atom (see Fig. 8a), at which the loop of overlapping orbitals is compressed (coarctated). Analogous to pericyclic reactions, the transition state of coarctate reactions is stabilized by orbital interactions, which can be either Hückel or Möbius type. The transition state leading to **IM3** is stabilized by a Möbius type eight-electron interaction and the coarctation of the orbital loop is at the P atom (see Fig. 8b). Coarctate reactions can be further classified as real or pseudo-coarctate reactions. In the latter, the electron delocalization includes a disconnection. Pseudo-coarctate reactions typically exhibit planar transition states and low activation barriers. Since the transition state TS_(_**_IM2_**_→_**_IM3_**_)_ is almost planar and the barrier is very small, we propose that this reaction has pseudo-coarctate character. This is further supported by the ACID (anisotropy of induced current density) plot^30^ (Fig. 8c), which shows a disconnection in the topology of the delocalized electrons.^31^ Similarly, the fragmentation of 3-azidoacrylaldehyde to isoxazol and dinitrogen has been described and classified as a pseudo-coarctate process.^32^

## Conclusion

In conclusion, we have presented a one-pot synthesis for substituted 1,2,4-oxadiphospholes, utilizing Na(OCP) as the phosphorous source. The reaction is remarkably selective and atom economic with respect to phosphorous and excellent yields can be obtained. The (OCP)^–^ anion was found to react as a nucleophile and P^–^ transfer reagent. The stepwise reaction mechanism was deciphered using low temperature ^31^P-NMR spectroscopy, kinetic measurements and theoretical calculations. Several [2 + 2] cycloaddition products were observed as intermediates using ^31^P NMR spectroscopy and identified with the help of chemical shift calculations, which demonstrate the performance of these theoretical methods. The formation of another, not yet observed, intermediate was made plausible through the reaction of Na(OCP) with a thioacyl chloride which gave a stable sodium 1,2,4-thiadiphosphol-3-olate. As observed previously in reactions with isocyanates^13^ and carbon dioxide,11*c* a remarkable feature in the reactions between (OCP)^–^ salts and heterocumulenes is the equilibrium, X=Y=Z + (OCP)^–^ ⇆ [XY(PCO)Z]^–^, which indicates the stability of the (OCP)^–^ anion with respect to its addition products. The final formation of the oxadiphosphole ring proceeds in a concerted, pseudo-coarctate reaction step. This transformation is reminiscent of reactions seen with α,β-unsaturated organic azides and indicates a possible resemblance between the isovalence electronic (OCP)^–^ and (N_3_)^–^ anions.

## Supplementary Material

Supplementary information

Crystal structure data
