# Supplementary material for: The reactivity of acyl chlorides towards sodium phosphaethynolate, Na(OCP): a mechanistic case study
Source: Chem Sci. 2016 Jun 17;7(9):6125–31. doi: 10.1039/c6sc01269h (PMC6024182; doi:10.1039/c6sc01269h)
Supplement: Supplementary file 1 [file SC-007-C6SC01269H-s001.pdf]

Electronic Supplementary Material (ESI) for Chemical Science.
This journal is © The Royal Society of Chemistry 2016

Supporting Information

1. Experimental details

1.1 Materials and methods

All reactions were performed using standard Schlenk techniques under a dry argon atmosphere. Sensitive chemicals were stored and weighed in a glove box under argon atmosphere. All solvents were purified and dried by standard methods. [Na(OCP)(dioxane)$_{2.5}$] was synthesized by literature methods.[1-2] 2,4,6-trimethylbenzylthioacid chloride was prepared based on literature procedures as well.[3-5] All other reagents were used without further purification as received from commercial suppliers. NMR spectra were recorded on BRUKER 250, 300, 400 or 500 MHz spectrometers. Deuterated solvents were further dried and purified prior to use. Chemical shifts are reported in ppm relative to $SiMe_4$ and 85% H_3PO_4 for ^{1}H, ^{13}C and ^{31}P respectively; Coupling constants are given in Hz. IR spectra were recorded on a Perkin-Elmer-Spectrum 2000 FT-IR spectrometer. The IR spectra in solid state were collected using an ATR device under inert atmosphere. The absorption bands are described as follows: very strong (vs), strong (s), medium (m) and weak (w); br stands for broad. Elemental analyses were performed at the microanalysis laboratory of the ETH Zurich. X-ray diffraction measurements were performed on either an Oxford XCalibur or a Bruker APEX-II single crystal diffractometer. UV/Vis spectra were recorded on a UV/VIS Lambda 19 spectrometer.

1.2 Experimental procedures

1.2.1 Synthesis of 3

A solution of 2,4,6-trimethylbenzoyl chloride (0.9 g, 4.96 mmol) in THF (10 mL) was slowly added dropwise under vigorous stirring at -78°C to a solution of [Na(OCP)(dioxane)$_{2.5}$] (1.5 g, 4.96 mmol) in THF (20 mL). The reaction mixture was allowed to warm to room temperature within 60 min. The reaction solution turned from colorless to yellow and a fine precipitate (sodium chloride) was formed. The reaction solution was stirred for another 60 minutes at room temperature and subsequently filtered to remove the sodium chloride. The solvent was removed under reduced pressure and a yellow solid was obtained. It was washed with copious amounts of

hexane to remove the excess of 2,4,6-trimethylbenzoyl chloride. The resulting fine yellow powder was dried *in vacuo*. Yield: 1.3 g (68%)

EA(%) calculated for $C_{21}H_{22}O_3P_2$: C 65.63, H 5.77%; found: C 63.19, H 5.90; The low carbon value is presumably due to trace amounts of non-combustible solid residues.

MP: 140°C

^{31}P -NMR (CD_2Cl_2, 121.49 MHz): δ (ppm) = 253.2 (d, $^2J_{PP}$ = 45.6 Hz, P1), 111.5 (d, $^2J_{PP}$ = 45.6 Hz, P2);

^{13}C-NMR (CD_2Cl_2, 125.8 MHz): δ (ppm) = 207.4 (dd $^1J_{PC}$ = 102.3 Hz, $^1J_{PC}$ = 55.1 Hz, C1), 203.5 (dd, $^1J_{PC}$ = 49.2 Hz, $^2J_{PC}$ = 4.3 Hz, C2), 169.4 (d, $^4J_{PC}$ = 6.4 Hz, C=O), 141.0 (s, 1C, C8), 138.8 (s, 2C, C5/C11), 137.6 (s, 1C, C17), 137.0 (d, J_{PC} = 5 Hz, 2C, C14/C20), 133.5 (dd, $^2J_{P2C}$ = 18.8 Hz, $^3J_{P1C}$ = 1.8 Hz, 1C, C13), 129.1 (s, 2C, C16/C19), 128.7 (s, 2 C, C7/C10), 126.5 (d, J_{PC} = 2.5 Hz, 1C, C4), 20.8-20.7 and 20.3-20.1 (6 C_{methyl}, C6/C9/C10/C15/C18/C21);

^{1}H-NMR (C_6D_6, 500.2 MHz): δ (ppm) = 6.85 (s, 2 H, H16/H19), 6.68 (s, 2 H, H7/H10), 2.35 (s, 6 H, H6/H12), 2.30 (s, 6 H, H15/H21), 2.22 (s, 3 H, H18), 2.10 (s, 3 H, H9);

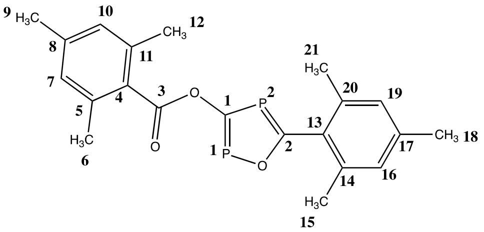

IR(cm^{-1}) *powder*: 1788 (m), 1732 (m), 169 (m, C=O), 1607 (m), 1442 (br m), 1421 (br m), 1377 (m), 1246 (m), 1213 (s), 1177 (s, C−O in ring), 1147 (s, C_{ring}−O_{ester}), 1059 (s), 970 (s), 946 (s), 849 (s), 698 cm^{-1} (s);

UV/VIS (hexane) λ_{max} = 247, 300 nm;

^{31}P- NMR shifts of further oxadiphospholes obtained from Na(OCP) with:

o-toluoyl chloride

^{31}P NMR (162.0 MHz, C_6D_6, 25°C) δ (ppm) = 248.8 (d, $^2J_{PP}$ = 45 Hz), 108.9 (d, $^2J_{PP}$ = 45 Hz);

2,6-difluorobenzoyl chloride

^{31}P NMR (121.5 MHz, THF, 25°C) δ (ppm) = 248.2 (d, $^2J_{PP}$ = 49 Hz), 122.8 (td, $^2J_{PP}$ = 49 Hz, $^4J_{PF}$ = 76 Hz);

Benzoyl chloride

^{31}P NMR (101.3 MHz, THF, 25°C) δ (ppm)= 260.1 (P1), 146.8 (P2);

Pivaloyl chloride

^{31}P NMR (162.0 MHz, THF, 25°C) isomer 1 (60%): δ (ppm) = 255.6 (d, $^{2}J_{PP}$ = 46 Hz), 90.3 (d, $^{2}J_{PP}$ = 46 Hz); isomer 2 (40%): δ (ppm) = 260.7 (d, $^{2}J_{PP}$ = 44 Hz), 91.5 (d, $^{2}J_{PP}$ = 44 Hz); Repetition of the ration delivered different ratios of the two isomers.

Cyclohexanecarbonyl chloride

^{31}P NMR (162.0 MHz, THF, 25°C) isomer 1 (85%): δ (ppm) = 254.4 (d, $^{2}J_{PP}$ = 45 Hz), 91.3 (d, $^{2}J_{PP}$ = 45 Hz); isomer 2 (15%): δ (ppm) = 258.5 (d, $^{2}J_{PP}$ = 43 Hz), 92.4 (d, $^{2}J_{PP}$ = 43 Hz);

1.2.2 Synthesis of [Na(5)(DME)$_2$]

2,4,6-trimethylbenzylthioacid chloride (1.0 g, 5 mmol) was dissolved in 10 mL of THF and was added dropwise at −78 °C to a solution of [Na(OCP) (dioxane)$_{2.5}$] (2.7 g, 9 mmol) in 20 mL THF. The reaction mixture was stirred at −78 °C for 1 hour and then allowed to warm to room temperature. The solvent was removed under reduced pressure and the obtained residue washed with hexane. Subsequently, the orange solid was dried *in vacuo* to yield 1.6 g of an orange solid, which was not analytically pure but clean enough for further reactions. Single crystals were obtained from a DME solution layered with MeO^{t}Bu at −30 °C. Single crystalline yield calculated for [Na(**5**)(DME)$_2$] was 280 mg (11%). MF: $C_{19}H_{31}Na_1O_5P_2S_1$.

MP: 278 °C (decomposition);

^{31}P{^{1}H}-NMR (THF-d8, 121.5 MHz): δ (ppm) =210.0 (d, $^{2}J_{P,P}$ = 38.5 Hz), 164.1 (d, $^{2}J_{P,P}$ = 38.5 Hz);

^{13}C{^{1}H}-NMR (THF-d8, 75.4 MHz): δ (ppm) =250 (dd, $^{1}J_{C,P}$ = 87.1 Hz, $^{1}J_{C,P}$ = 67.0 Hz), 181.24 (dd, $^{1}J_{C,P}$ = 55.5 Hz, $^{2}J_{C,P}$ = 7.7 Hz), 135.7 (dd, $^{2}J_{C,P}$ = 15.81 Hz, $^{2}J_{C,P}$ = 5.9 Hz), 134.1 (d, $^{3}J_{C,P}$ =2.0 Hz), 125.7 (s), 134.1 (s), 17.8 (d, $^{4}J_{C,P}$ = 0.9 Hz), 18.3 (s);

^{1}H-NMR (THF-d8, 300 MHz): δ (ppm) =6.8 (s, 2H, CH), CH_3 0.42 (s, 6H, CH_3), 0.53 (s, 3H, CH_3);

IR(cm^{-1}) *powder*: 2915.68, 1450.79, 1259.32, 1084.74, 1030.72, 848.31, 802.24; UV/VIS (THF) λ_{max} = 422, 340 nm

1.2.3 Synthesis of 7

[Na(**5**)(DME)$_2$] (150 mg, 0.33 mmol) was dissolved in 5 mL THF and was cooled to 0 °C. To this solution 2,4,6-trimethylbenzoyl chloride (75 mg, 0.4 mmol) diluted in 5 mL THF was added drop wise. The reaction was stirred for 2 h at room temperature and the volatiles were removed under reduced pressure. The solid was dissolved in toluene and filtered over Celite. The volatiles were removed again and the product was washed with hexanes. The hexane washing solution was placed at −30 °C in the freezer. The solid was dried under reduced pressure yielding 40 mg (30%) of a yellow solid. From the washing solution another 19 mg (14 %) of product were isolated as an off white solid. Total yield was 59 mg (44 %). The product was recrystallized from a saturated toluene solution at −30 °C to obtain single crystals suitable for X-ray analysis, which confirmed the expected connectivity of the atoms.

EA(%) calculated for $C_{21}H_{22}O_2P_2S$: C, 62.99; H, 5.54; found: C, 61.66; H, 5.61;

MP: 116 °C;

$^{31}P\{^1H\}$-NMR (CD_2Cl_2, 121.5 MHz): δ (ppm) = 229.13 (d, $^2J_{P,P}$ = 67.0 Hz), 232.6 (d, $^2J_{P,P}$ = 67.0 Hz);

^1H-NMR (CD_2Cl_2, 300 MHz): δ (ppm) = 6.89 (s, 4H, CH_{arom}), 2.34 (s, 6H, CH_3), 2.24 (m, 6H, CH_3), 2.04 (s, 6H, CH_3);

$^{13}C\{^1H\}$-NMR (CD_2Cl_2, 75.47 MHz): δ (ppm) = 211.6 (dd, $^1J_{PC}$ = 100.0 Hz, $^1J_{PC}$ = 61.1 Hz, C1), 185.1 (dd, $^1J_{PC}$ = 49.8 Hz, $^2J_{PC}$ = 8.6 Hz, C2), 167.5 (d, $^4J_{PC}$ = 7.7 Hz, C=O), 140.5 (s), 137.8 (s), 136.3 (s), 136.0 (d, J_{PC} = 4.7 Hz), 132.2 (dd, $^2J_{P2C}$ = 19.3 Hz, $^3J_{P1C}$ = 4.4 Hz), 128.3 (s), 127.7 (s), 126.7 (s), 20.2 (s, CH_3), 20.01 (s, CH_3), 19.8 (s, CH_3), 19.5 (s, CH_3);

IR(cm^{-1}) *powder*: 1701.7 (C=O) band;

UV/VIS (THF) λ_{max} = 324 (shoulder), 264 nm;

LT-NMR kinetic investigation on the formation mechanism of 3

A solution of [NaOCP (dioxane)$_{2.5}$] (45 mg, 0.15 mmol) in THF (0.3 mL) was prepared in an NMR tube. The NMR tube was cooled to −60°C and under inert atmosphere a solution of 2,4,6-trimethylbenzoyl chloride (30 mg, 0.16 mmol) in THF (0.2 mL) was added slowly dropwise at -60°C. The cold NMR tube was transferred into the precooled (−35°C) 400 MHz NMR machine. The reaction was followed for

117 hours by ^{31}P-NMR spectroscopy. The quantitative NMR measurement was performed employing the methodology in reference 2. The data set was analysed using the Matlab programme (MATLAB and Statistics Toolbox Release 2010b, The MathWorks, Inc., Natick, Massachusetts, United States).

2. UV/Vis spectra of compounds 3, Na(5) and 7

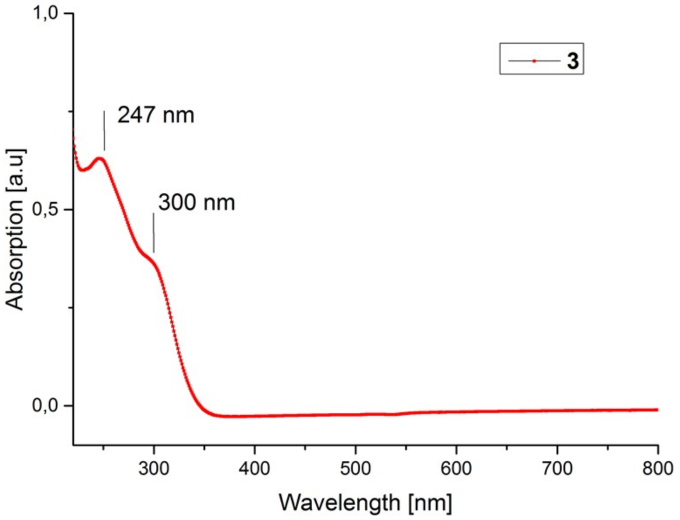

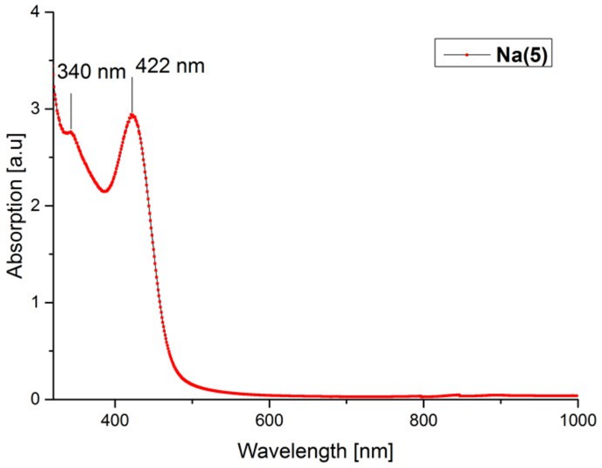

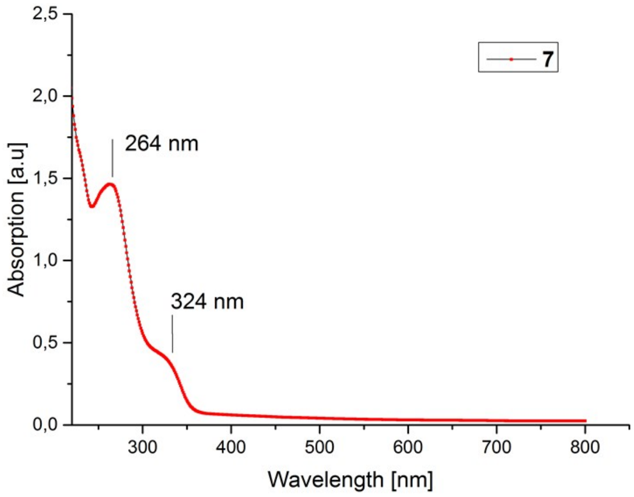

2,5
2,0
1,5
1,0
0,5
0,0
Absorption [a.u]
264 nm
324 nm
7
300
400
500
600
700
800
Wavelength [nm]

3. X-ray structure analyses

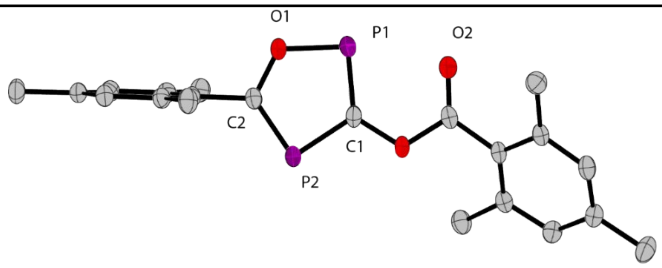

Table S1 Crystal data and structure refinement for **3**

Identification code	CCDC 986673
Formula weight	384.33
Temperature/K	120(2)
Crystal system	triclinic
Space group	P-1
a/Å	7.1039(5)
b/Å	8.2133(6)
c/Å	17.3480(12)
α/°	102.227(6)
β/°	90.177(6)
γ/°	90.407(6)
Volume/Å^3	989.20(12)
Z	2
ρ_{calc}mg/mm^3	1.290
m/mm^{-1}	0.237
F(000)	404.0
Crystal size/mm^3	0.41 × 0.23 × 0.21
2Θ range for data collection	6.06 to 58.04°
Index ranges	$-9 \le h \le 9, -10 \le k \le 11, -23 \le l \le 23$
Reflections collected	19150
Independent reflections	4744[R(int) = 0.0561]
Data/restraints/parameters	4744/0/242
Goodness-of-fit on F^2	1.105
Final R indexes [I>=2σ (I)]	$R_1 = 0.0476$, $wR_2 = 0.1280$
Final R indexes [all data]	$R_1 = 0.0539$, $wR_2 = 0.1319$
Largest diff. peak/hole / e Å^{-3}	0.40/-0.45

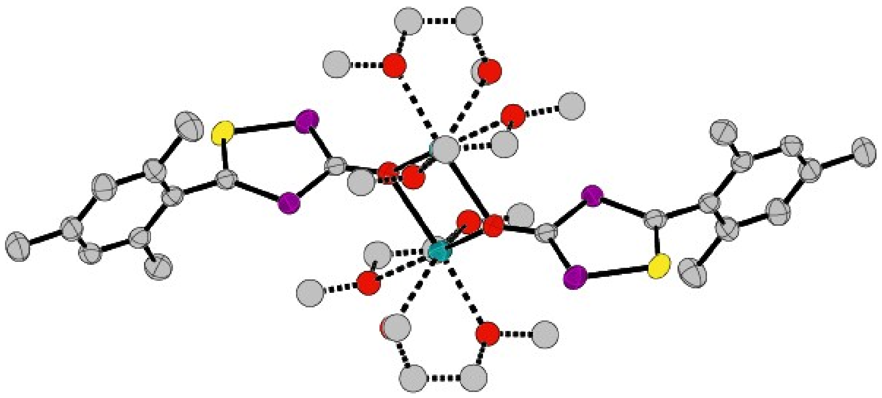

**Table S2:** Crystal data and structure refinement for **5**.	
Identification code	CCDC 1454789
Empirical formula	$C_{38}H_{62}Na_2O_{10}P_4S_2$
Formula weight	912.86
Temperature/K	100.0
Crystal system	monoclinic
Space group	$P2_1/n$
a/Å	9.4422(4)
b/Å	10.6570(4)
c/Å	24.5794(10)
α/°	90.00
β/°	100.2517(7)
γ/°	90.00
Volume/Å^3	2433.83(17)
Z	2
ρ_{calc}mg/mm^3	1.246
m/mm^{-1}	0.307
F(000)	968.0
Crystal size/mm^3	0.28 × 0.18 × 0.15
Radiation	MoKα (λ = 0.71073)
2Θ range for data collection	3.36 to 52.74°
Index ranges	-11 ≤ h ≤ 10, -13 ≤ k ≤ 13, -30 ≤ l ≤ 30
Reflections collected	18542
Independent reflections	4974 [R_{int} = 0.0265, R_{sigma} = 0.0255]
Data/restraints/parameters	4974/61/274
Goodness-of-fit on F^2	1.065
Final R indexes [I>=2σ (I)]	R_1 = 0.0501, wR_2 = 0.1225
Final R indexes [all data]	R_1 = 0.0612, wR_2 = 0.1310
Largest diff. peak/hole / e Å^{-3}	0.76/-0.63

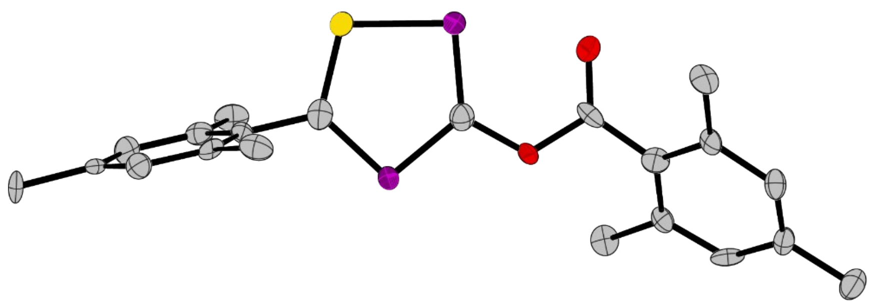

**Table S3:** Crystal data and structure refinement for **7**.	
Identification code	CCDC 1454790
Empirical formula	$C_{21}H_{22}O_2P_2S$
Formula weight	400.38
Temperature/K	110.6(3)
Crystal system	triclinic
Space group	P-1
a/Å	7.1716(4)
b/Å	8.4029(7)
c/Å	17.181(2)
α/°	80.076(9)
β/°	88.792(8)
γ/°	86.067(6)
Volume/Å^3	1017.46(17)
Z	2
ρ_{calc}g/cm^3	1.307
μ/mm^{-1}	0.329
F(000)	420.0
Crystal size/mm^3	0.14 × 0.38 × 0.36
Radiation	MoKα (λ = 0.71073)
2Θ range for data collection/°	5.694 to 49.424
Index ranges	-8 ≤ h ≤ 8, -9 ≤ k ≤ 9, -20 ≤ l ≤ 17
Reflections collected	7325
Independent reflections	3451 [R_{int} = 0.0619, R_{sigma} = 0.0977]
Data/restraints/parameters	3451/0/241
Goodness-of-fit on F^2	1.026
Final R indexes [I>=2σ (I)]	R_1 = 0.1680, wR_2 = 0.4350
Final R indexes [all data]	R_1 = 0.1856, wR_2 = 0.4428
Largest diff. peak/hole / e Å^{-3}	1.85/-0.76

4. Theoretical calculations

The calculations have been performed using the Gaussian 09 code.[6] The geometries were fully optimized and second order derivatives were obtained to check whether the optimized structures are minima (positive eigenvalues) or transition states (one negative eigenvalue). The anisotropy of induced current density calculations have

been carried out with the program AICD-2.0.0 offered by Prof. Herges.[7] The NMR chemical shift values were calculated applying the method in reference 8.

Geometries and total energies

IM2

CCSD(T)= -1060.8199389

C	-1.01621	0.13334	-0.18173
P	-0.89217	0.16552	1.64625
C	0.89749	0.19957	1.92477
C	1.33395	0.1646	3.39262
O	-2.09881	0.14907	-0.7527
P	0.67852	0.06369	-1.23051
C	-0.07819	-0.46321	-2.65288
O	-0.42154	-0.90177	-3.68856
O	1.80282	0.25422	1.06951
H	1.96462	1.03913	3.59933
H	1.946	-0.73207	3.55768
H	0.48749	0.15458	4.08887

TS

CCSD(T)= -1060.8143888

C	0.000000	0.000000	0.000000
P	0.000000	0.000000	1.865242
O	1.898525	0.000000	1.901961
C	2.540560	-0.027189	0.763864
C	4.045581	-0.042436	0.901228
P	1.709386	-0.041538	-0.763833
O	-0.999705	0.016752	-0.735121
C	-2.052961	-0.492910	1.699969
O	-2.953019	-1.090516	2.114596
H	4.361349	-0.925142	1.475077
H	4.382565	0.844890	1.455540
H	4.539653	-0.059048	-0.075839

IM3

CCSD(T)= -1060.8282481

C	-0.8531619	0.2459777	-0.3399161
P	-1.3483988	-0.1477988	1.4375335
C	0.281651	-0.1351639	2.0122947
C	0.7368483	-0.3845275	3.4255686
O	-1.6884231	0.3346492	-1.2644709
P	0.9333221	0.4775386	-0.4993704
C	0.4466143	0.184352	-3.6651716
O	0.3911107	-0.837808	-4.1706379
O	1.2926252	0.1196628	1.1808563
H	1.2836464	0.487196	3.814646
H	1.4226753	-1.2437057	3.4665349
H	-0.1143837	-0.5854485	4.0837396

Literature:

1. F. F. Puschmann, D. Stein, D. Heift, C. Hendriksen, Z. A. Gál, H.-F. Grützmacher and H. Grützmacher, *Angew. Chem. Int. Ed.*, 2011, 50, 8420-8423.
2. D. Heift, Z. Benkő and H. Grützmacher, *Dalton Trans.*, 2014, 43, 831-840.
3. R. Mayer and S. Scheithauer, *Chem. Ber.*, 1965, 98, 829-837.
4. S. Scheithauer and R. Mayer, *Chem. Ber.*, 1965, 98, 838-843.
5. R. Mayer and S. Scheithauer, *Journal für Praktische Chemie*, 1963, 21, 214-224.
6. G. W. T. M. J. Frisch, H. B. Schlegel, G. E. Scuseria, M. A. Robb, J. R. Cheeseman, G. Scalmani, V. Barone, B. Mennucci, G. A. Petersson, H. Nakatsuji, M. Caricato, X. Li, H. P. Hratchian, A. F. Izmaylov, J. Bloino, G. Zheng, J. L. Sonnenberg, M. Hada, M. Ehara, K. Toyota, R. Fukuda, J. Hasegawa, M. Ishida, T. Nakajima, Y. Honda, O. Kitao, H. Nakai, T. Vreven, J. A. Montgomery, Jr., J. E. Peralta, F. Ogliaro, M. Bearpark, J. J. Heyd, E. Brothers, K. N. Kudin, V. N. Staroverov, R. Kobayashi, J. Normand, K. Raghavachari, A. Rendell, J. C. Burant, S. S. Iyengar, J. Tomasi, M. Cossi, N. Rega, J. M. Millam, M. Klene, J. E. Knox, J. B. Cross, V. Bakken, C. Adamo, J. Jaramillo, R. Gomperts, R. E. Stratmann, O. Yazyev, A. J. Austin, R. Cammi, C. Pomelli, J. W. Ochterski, R. L. Martin, K. Morokuma, V. G. Zakrzewski, G. A. Voth, P. Salvador, J. J. Dannenberg, S. Dapprich, A. D. Daniels, Ö. Farkas,

J. B. Foresman, J. V. Ortiz, J. Cioslowski and D. J. Fox, Gaussian 09, Revision A.02, *Gaussian Inc. , Wallingford CT* 2009.

7. a) R. Herges and D. Geuenich, *J. Phys. Chem. A* 2001, **105**, 3214-3220; b) D. Geuenich, K. Hess, F. Kohler and R. Herges, *Chem. Rev.* 2005, **105**, 3758-3772.

8. D. Heift, Z. Benkő and H. Grützmacher, *Dalton Trans.* 2014, **43**, 5920-5928.
